# The Impact of COVID-19 on Cognitive Function

**DOI:** 10.1093/geroni/igaf122.2310

**Published:** 2025-12-31

**Authors:** Adriana Hernandez, Jeffrey Stokes

**Affiliations:** University of Massachusetts Boston, Boston, Massachusetts, United States; University of Massachusetts Boston, Boston, Massachusetts, United States

## Abstract

The present study examined the association between COVID-19 infection and cognitive function among adults aged 50 and older. Specifically, we investigated whether individuals who contracted COVID-19 in 2020 experienced greater declines to cognitive functioning by 2022 compared to individuals who did not contract COVID-19. This study used data from the 2020 and 2022 wave of The Health and Retirement Study. The sample consisted of 7,349 participants aged 50 and older, with 6,989 participants reporting “no” to testing positive for COVID-19 and 360 participants reporting “yes.” Cognitive function was determined using episodic memory scores from the HRS-adapted version of the Telephone Interview for Cognitive Status (TICS). A series of logistic and linear regression models were employed to estimate the association between cognitive function and COVID-19 infection. Results indicated that individuals who tested positive for COVID-19 showed greater declines in memory over two years than people who tested negative for COVID-19. However, COVID-19 was not randomly distributed and was associated with factors that also lead to declines in cognition such as: age, depressive symptoms, and employment status. Therefore, cognitive decline reflected other causal factors rather than a direct association with COVID-19 infection.

